# Impact of Cerebral Visual Impairments on Motor Skills: Implications for Developmental Coordination Disorders

**DOI:** 10.3389/fpsyg.2016.01471

**Published:** 2016-10-04

**Authors:** Sylvie Chokron, Gordon N. Dutton

**Affiliations:** ^1^Unité Fonctionnelle Vision and Cognition, Fondation Ophtalmologique RothschildParis, France; ^2^Laboratoire de Psychologie de la Perception, UMR 8242, Centre National de la Recherche Scientifique – Université Paris-DescartesParis, France; ^3^Department of Vision Science, Glasgow Caledonian UniversityGlasgow, UK

**Keywords:** children, cerebral visual impairment (CVI), occipital lobe, learning disorders, developmental coordination disorder (DCD), cerebral palsy (CP)

## Abstract

Cerebral visual impairment (CVI) has become the primary cause of visual impairment and blindness in children in industrialized countries. Its prevalence has increased sharply, due to increased survival rates of children who sustain severe neurological conditions during the perinatal period. Improved diagnosis has probably contributed to this increase. As in adults, the nature and severity of CVI in children relate to the cause, location and extent of damage to the brain. In the present paper, we define CVI and how this impacts on visual function. We then define developmental coordination disorder (DCD) and discuss the link between CVI and DCD. The neuroanatomical correlates and aetiologies of DCD are also presented in relationship with CVI as well as the consequences of perinatal asphyxia (PA) and preterm birth on the occurrence and nature of DCD and CVI. This paper underlines why there are both clinical and theoretical reasons to disentangle CVI and DCD, and to categorize the features with more precision. In order to offer the most appropriate rehabilitation, we propose a systematic and rapid evaluation of visual function in at-risk children who have survived preterm birth or PA whether or not they have been diagnosed with cerebral palsy or DCD.

## Introduction: From Visual Perception to Action

In the course of child development, vision precedes action, and during its 1st months the baby experiences a visual relationship with the outside world before being able to voluntarily act within it (Itier and Batty, 2009). Indeed, as Lipsitt and Spiker (1967) point out, the newborn is both non-verbal and motorically immature with a limited behavioral repertoire although its visual perception is already at work (Chokron and Streri, 2012; de Hevia et al., 2014). As Braddick and Atkinson (2013) explain, infants aged between 5 and 18 months show an almost compulsive response to reach out, grasp, and manipulate any small object placed in front of them. As these authors discuss, this is a striking motor behavior, but it is also a visual behavior reliant upon the dorsal and ventral streams of the visual system (Milner and Goodale, 2008).

The development and improvement of perceptual and motor skills such as spatial orientation, coordination (hand–eye, foot–eye, hand–foot–eye coordination), balance, and body awareness are dependent on an effective visual system as well as good eye muscle control (Willoughby and Polatajko, 1995; Cheatum and Hammond, 2000; Coetzee and Pienaar, 2013). In this way, if there is any defective input of information by way of the visual system, the reaction of the motor output to such information will also be defective, leading to visuo-motor or motor deficiencies and poor concentration (Peens and Pienaar, 2007; Coetzee and Pienaar, 2013).

For example, catch a ball. You see its details, you identify it, you distinguish it from surrounding objects, you choose it, you predict its vector, and you configure and move your hand to catch it. This multi-step process is remarkable, as the total requisite ‘computing process’ is performed within the brain (Machado et al., 2008). The analysis of detail, (in terms of clarity, contrast, and color) is accomplished in the occipital lobes (Dutton, 2003; Peelen and Kastner, 2014). Recognition of its identity is achieved by the temporal lobes (Milner and Goodale, 2008; Peelen and Kastner, 2014). Its initial location and form are mapped in the parietal lobes (Sewards, 2011). Its vector is appreciated by combined activity in the middle temporal and posterior parietal lobes (Brozzoli et al., 2014). The predicted location of the ball is provided by prior experiential learning including oculomotor, motor, perceptual and spatial experience (mostly in the frontal and parietal lobes; Machado et al., 2008; Brozzoli et al., 2014). The requisite temporary non-conscious internal 3D emulation of the visual scene is created by the occipital and posterior parietal lobes (Chokron et al., 2004; Dulin et al., 2008), and the moment-to-moment 3D coordinates of the shape and location of the ball reach the motor cortex which, supported by the timing system in the cerebellum, the overall balance system, and the reflex motor support systems in the brain stem and thalamus, brings about the requisite finely tuned action to catch the ball, while the choice of catching the ball was made in the frontal lobes (Machado et al., 2008).

Disruption in any part of this complex visuo-motor system, as we will discuss below, disturbs this mundane act, rendering it difficult or impossible.

## Cerebral Visual Impairments (CVI)

### Definition

Cerebral visual impairment (CVI) relates to damage or malfunction of the visual pathways or visual centers in the brain, including the lateral geniculate bodies, the optic radiations, the occipital cortices and the visual associative areas, as well as tectum and thalamus (Barton, 2011; Lehman, 2012). The features may be accentuated by associated disorders of eye movement control (Fazzi et al., 2009; Boot et al., 2010; Ortibus E. L. et al., 2011). CVI is not a single diagnosis. As developmental coordination disorder (DCD) is an umbrella term for several types of motor deficit (Pearsall-Jones et al., 2010), CVI is an umbrella term for all types of visual impairment due to brain damage or dysfunction. Each affected child has their own unique clinical picture which needs to be identified and individually profiled.

### How Visual Disturbances Can Result from Cerebral Visual Impairments

It is clear that vision cannot simply be reduced to the mere capacity to detect and resolve a visual stimulus. Seeing encompasses an ensemble of actions to interact with and learn about the outside world: recognizing one’s loved ones and the environment; invoking the mirror neurone system to imitate gestures and actions so as to acquire the skills needed to communicate and to manipulate objects; being able to visually gage accurate reach and grasp, and being able to visually navigate accurately through space while avoiding obstacles. Seeing also means paying visual attention to one’s surroundings, being able to recognize, identify and select one object from amongst several others, and being able to understand a complex visual scene, an ambiguous figure or a painting in the context of prior visual experience and knowledge. Likewise, seeing also facilitates recognition of written language and other symbols, guiding the movement of the writing hand, organizing handwriting on a page, or spatially arranging the steps needed to perform a calculation. Seeing includes perceiving and decoding the emotions of others, to reciprocate smiles and other facial expressions, and recognizing familiar faces, pets or places and to respond accordingly, often with mirrored behaviors. Over the course of a child’s development, vision fundamentally facilitates learning, leading to knowledge, skills and traits that will shape the child’s personality and cognition. If children with CVI are not identified and appropriate measures taken to ensure that all educational input is visible or rendered accessible by alternative means, they cannot learn within any domain limited by their unique pattern of visual impairment.

**FIGURE 1:**
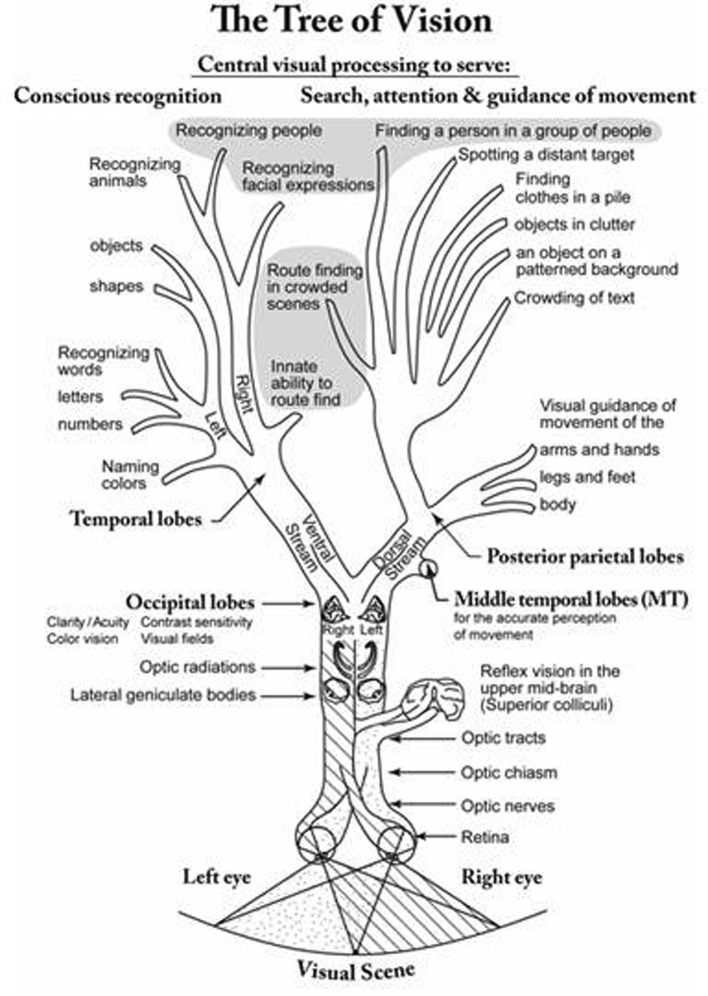
**Stylised diagram resembling a tree denoting the visual pathways and the visual capacities they serve**.

Lack of learning due to failure to cater for CVI can resemble a primary intellectual deficit (see for review and discussion, Lueck and Dutton, 2015). Behaviors that are adaptive to enable the child to cope, or are reactive owing to the stress caused by certain environments or conditions exceeding mental processing capacity, can resemble a range of disorders such as ASD and ADHD, but rapid developmental progress when appropriate measures are taken, belies such interpretations (Pawletko et al., 2015). Now better understood, characterized and diagnosed, the impairments of vision, visual perception and cognition due to CVI (**Figure 1**), are currently being studied to provide understanding of how they contribute to the complex puzzle of disorders of learning, developmental coordination, and social-interaction (Lehman, 2012; Lueck and Dutton, 2015).

Below, we present the epidemiology of CVI before describing how spatial as well as recognition deficits observed in children with CVI significantly alter the origin, motivation and planning of actions, as well as visuo-motor coordination.

### Epidemiology

Recent advances in the treatment of sight threatening pediatric eye conditions including retinopathy of prematurity, cataract, and glaucoma, have diminished the prevalence of ocular visual impairment, while in industrialized nations, advances in neonatal medicine have increased survival rates of both prematurely born infants, and those who develop neurologic lesions during or shortly after birth. This has led to neurologic disorders becoming the commonest cause of impairment of vision in children in industrialized nations (Dutton and Bax, 2010; Kong et al., 2012). Yet the International Classification of Disease (ICD 10) fails to include this diagnostic category as a cause of blindness and visual impairment. This can result in failure of governments and administrative bodies to recognize this multifaceted condition. Current estimates suggest that as many as 3 to 4% of children aged between 4 and 6 years (i.e., approximately one student per kindergarten class) may have an identifiable visual or/and attentional deficit as a sequel to a possible neurologic lesion or dysfunction sustained around the time of birth (Cavézian et al., 2010). Unrecognized visual dysfunctions due to CVI can be inappropriately categorized as Learning Difficulties (Chokron and Démonet, 2010; Lueck and Dutton, 2015). Optic nerve hypoplasia and atrophy have previously been considered isolated diagnoses, but are now known to also be associated in some cases with cerebral visual pathway and cortex lesions (Zeki et al., 1992) that can also cause focal retinal ganglion cell layer atrophy (Lennartsson et al., 2014) due to retrograde trans-synaptic degeneration (Jacobson et al., 1997).

### Visual Impairments Due to Damage or Dysfunction of the Visual Pathways Behind the Optic Chiasm

**FIGURE 2:**
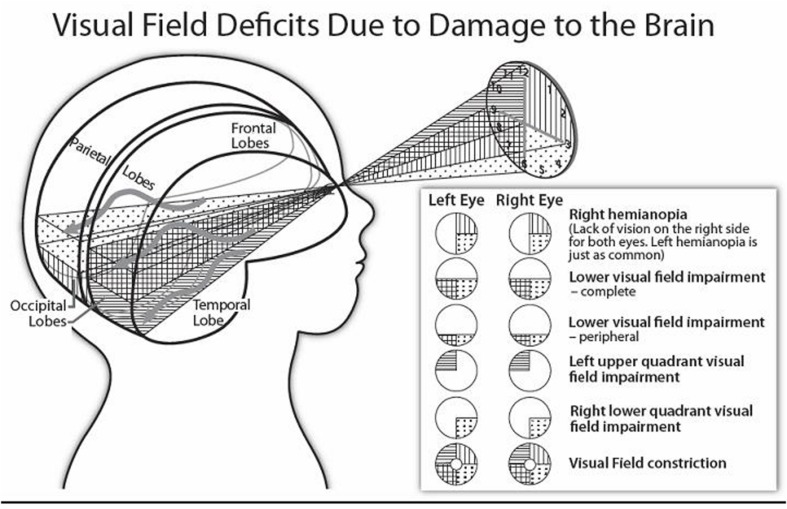
**Stylised diagram illustrating homonymous visual field disorders, and the approximate location of affected brain structures.** (The sinuous arrows denote the distinct pathways of the superior and inferior optic radiations around the lateral ventricles of the brain).

The central visual functions of visual acuity, contrast sensitivity, and color vision, as well as the peripheral visual fields can be aﬄicted by lesions or dysfunction affecting the optic tracts, lateral geniculate nuclei, optic radiations or primary visual cortices (Lueck and Dutton, 2015). As we present below, according to the location and extent of the post-chiasmatic lesion, the visual deficit varies considerably from a decrement in visual field or limited perceptual dysfunction to profound impairment of vision such as in cerebral (or cortical) blindness (i.e., lack of all visual function despite the integrity of the eyes; Lueck and Dutton, 2015). **Figure 2** illustrates how the visual fields can be affected.

#### Moderate to Mild Visual Impairments

We each ‘know’ that our vision is ‘normal.’ Children with CVI are no exception, especially if they are not aware that their low vision is responsible for diminishing their performance when compared with their peers. They are therefore not ‘symptomatic,’ but are often detected when their visual performances are identified by parents and carers as being sub-optimal. Visual acuity can be diminished or normal, while parental reports of even profound visual difficulties can sometimes be inappropriately dismissed by professionals, who erroneously equate ‘seeing’ with visual acuity.

#### Homonymous Lack of Vision

Homonymous lack of vision (in the same distribution in both eyes) commonly affects the quadrants of vision, either as complete absence or as impaired clarity of peripheral vision in the areas affected (Jacobson et al., 2006; Chokron et al., 2016).

#### Concentric Constriction of the Binocular Visual Field

Concentric constriction of the binocular visual field leads to tunnel vision, while bilateral lack of central vision manifests with central scotomas with preservation of peripheral vision. Some of these impairments are evident at birth, while others become apparent later in life (Guzetta et al., 2001a; Watson et al., 2007; Werth and Schadler, 2008). Acute or chronic progressive acquired disorders of the visual brain can present later in life.

#### Cerebral (Cortical) Blindness

Infants with cerebral blindness present early in life when they do not visually respond to their parents. Infants with severe damage to the occipital lobes and/or visual pathways may initially manifest lack of the blink reflex to light or visual threat, but commonly show slow progressive improvement in vision, or type 2 delayed visual maturation. Affected infants may not at first respond visually to movement, or to change between light and dark, but this tends to last only a few weeks; the child may eventually gain basic visual functions, and respond to high-contrast or moving visual stimuli. In some cases, when presented with visual and acoustic stimuli in the dark, these children may respond with ocular movements or by fixing their gaze, which they often cannot do when presented with visual stimuli—even high-contrast ones—in ambient light. Such children can show larger visual evoked potential signals in the dark than in light (Good and Hou, 2006).

Some children with little or no apparent vision (sometimes with cerebral palsy) may intermittently look toward movement to one side or the other, or both, and may occasionally open their mouths in response to a spoon approaching from the side although not being able to perceive (Dutton et al., 2014). This dissociation between absence of conscious perception and ability to react to an unseen stimulus is known as *blindsight* and was first defined by Weiskrantz et al. (1974). This type of blindsight may relate to intact tectal and pulvinar reflex visual functions (Tinelli et al., 2013). Other children, (like adults with cortical blindness), may occasionally respond to smiles (Boyle et al., 2005). This is known as affective blindsight (Celeghin et al., 2015).

We not infrequently see children who had cortical blindness at an early age, but whose CVI is only diagnosed several years later (Watson et al., 2007; Werth and Schadler, 2008). When they present with tunnel vision (perception within only a 10 to 20° central concentric area) or peripheral vision, together with other perceptual impairments such as simultanagnosia, optic ataxia, spatial orientation deficits, or impaired recognition of objects and/or faces, as described above. Unfortunately, the visual field defects can go unrecognized, partly because the child is unaware of his/her impairment and does not know that a “full” visual field extends horizontally over 180°, and partly because such defects are totally invisible unless sought (Pawletko et al., 2015). Visual impairment is truly a hidden disability.

### Deficits in Visual Cognition Due to Post-chiasmatic Pathology or Dysfunction

Lesions affecting the ventral stream pathways impair recognition of objects, people and route finding, while lesions of the dorsal stream pathways are accompanied by impairments that can interfere with visual exploration, visual attention (perhaps related to fewer items being mapped), spatial organization and representation, and visuo-motor coordination (see for complete semiological description of ventral and dorsal dysfunction, Dutton, 2015).

#### Ventral Stream Dysfunction

Visual recognition impairments, which in adult patients are collectively known as *visual agnosias*, result from damage to the occipito-temporal lobes and ventral stream pathways and are not linked to alterations in verbal function. Children with such deficits have difficulties interpreting what they see, but can still recognize what they access using their other senses (e.g., touch). These impairments most commonly affect recognition of images and objects (Pawletko et al., 2015); however, they can also affect recognition of faces, the ability to see and interpret facial expressions, and even spelling (for reviews of this topic, see Dutton and Bax, 2010; Lueck and Dutton, 2015).

At a more fundamental level of image interpretation, the capacity to estimate size and orientation of objects and lines can also be affected. Even if visual recognition deficits do not directly alter the planning of action in space (Goodale et al., 2008; Milner and Goodale, 2008), of course, the motivation to act toward unrecognized objects must be weaker than in typical developing children (Fazzi et al., 2015). In addition, as we present below, dorsal stream dysfunction leads to severe visuo-motor deficits.

#### Dorsal Stream Dysfunction

##### Balint syndrome

**FIGURE 3:**
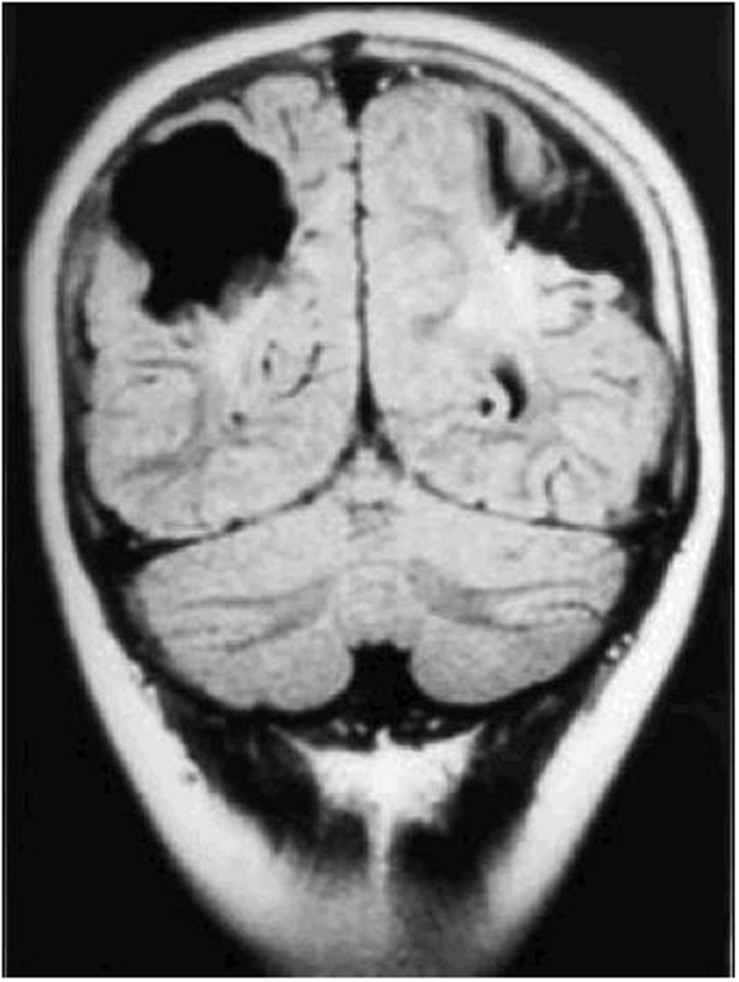
**Coronal CT scan of bilateral posterior parietal lobe scarring in a 10 years old boy with features of Balint syndrome.** (Reproduced by the author GN Dutton, from Gillen and Dutton, 2003.)

Damage to the parietal lobes results in defective three-dimensional mapping of the visual scene, with fewer of the surrounding entities in the scene being accessible for the frontal territory to accord attention to, culminating in simultanagnostic visual dysfunction (Barton, 2011). The impaired mapping also renders visual guidance of movement inaccurate (or optic ataxia), particularly when reaching to the side, as well as inaccuracy of, or inability to make visually guided saccades, despite evidence of an intact eye movement system (oculomotor apraxia). These features can occur singly or in combination. Severe variants of this condition comprise Balint syndrome (Rizzo and Vecera, 2002), while those that are less severe are commonly referred to as dorsal stream dysfunction (Macintyre-Beo et al., 2010). The superior optic radiations can also be affected, causing lower visual field impairment, which ranges between being complete, to solely rendering the feet invisible when walking. Impaired image resolution in the lower visual field (Jacobson et al., 2006) also impairs visual guidance of movement. **Figure 3** (MRI scan of bilateral posterior parietal lobe scarring) shows bi-parietal damage due to perinatal hypoxic ischaemic encephalopathy.

Children who are profoundly affected by quadriplegic cerebral palsy and intellectual disability may have severe posterior parietal damage culminating in possible inability to see more than one item (true simultanagnosia). This becomes evident when some affected children are seen to ‘wake up’ and become visually attentive for the first time, when enclosed by a monochromatic tent (Little and Dutton, 2015). This is consistent with a form of Balint syndrome masked by the motor and intellectual dysfunction (Gillen and Dutton, 2003). In the same way, periventricular leukomalacia (PVL) which is frequent in preterm children often leads to parietal damage and in this way to dorsal stream dysfunctions characterized by simultanagnosia as well as spatial and visuo-motor coordination deficits (Jacobson and Dutton, 2000; Jacobson et al., 2006; Fazzi et al., 2009).

##### Hemi-inattention and hemispatial neglect

**FIGURE 4:**
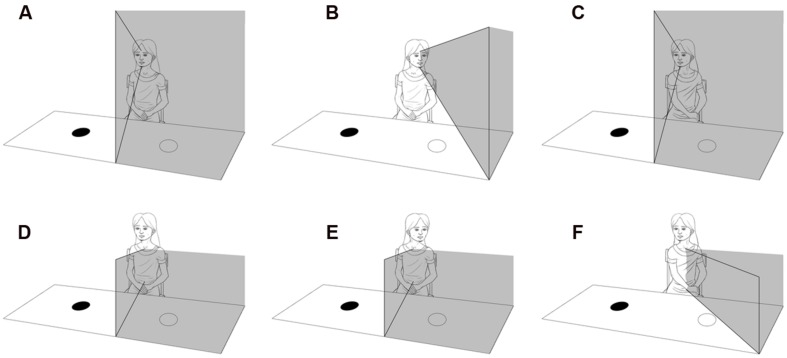
**Diagram illustrating the difference between hemianopia and visual neglect. (A)** Illustrates left hemianopia due to right occipital lobe damage that moves with rotation of the head and eyes **(B)**, but not rotation of the body **(C)**. **(D)** Illustrates left visual neglect due to damage of the right inferior posterior parietal lobe, that does not move with rotation of the head and eyes **(E)**, but does move with rotation of the body **(F)**.

As in adults, hemispatial neglect results from unilateral posterior parietal damage (Laurent-Vannier et al., 2001, 2006), it is more severe when left hemispatial attention is impaired by a right-sided lesion. It is characterized by difficulties in reacting to, or interacting with, stimuli presented on the side of the body contralateral to the lesion (Bartolomeo and Chokron, 2002). The affected child can behave as if half of their surrounding space does not exist (Laurent-Vannier et al., 2001, 2006). Visual auditory and tactile attention all tend to be deficient, with lack of searching, scanning, hearing, and motor function on the affected side. As presented in **Figure 4**, affected children tend to turn their body in the direction of their unaffected side, because the posterior parietal ‘map’ of the environment is body-centric and tends not to be compensated for by a head or eye turn (Chokron et al., 2007).

Children with CVI can also exhibit deficits in spatial organization and representation that can be evaluated by means of drawing and copying geometric shapes, arranging cubes, doing puzzles, and performing spatial reasoning exercises (i.e., visualizing an object, and then answering questions about this). Although frequently observed in clinical practice, deficits in the capacity to form mental imagery are not often reported in the literature on CVI in children. Nevertheless, such visual and spatial impairments are often discovered during clinical evaluation and are similar to what is observed in adult neglect patients including neglect dyslexia (Ellis et al., 1987; Leff et al., 2000; Chokron et al., 2004; Dulin et al., 2008).

### Dorsal Stream Dysfunction and Visuo-Motor Skills

Processing of visual information is paramount in executing and controlling *hand and body movement* (for reviews of this subject, see Shumway-Cook and Woollacott, 1995; Prablanc et al., 2003). As Molinaro et al. (2015) point out, vision and motor skills typically evolve together. As explained by these authors, vision for example enables children to recognize their caregivers, to know whether they are present or absent and motivates children to move toward them. In addition, Fraiberg (1977) hypothesized that lack of sight impairs the ability to build up a picture of the world, and without this, there is decreased incentive for developing voluntary skills. Indeed, vision is the first resource that children use not only to build a representation of the external world, including the permanence of objects, but also for postural control: only later do they employ tactile and vestibular information to this purpose (Guzetta et al., 2001b; Mazeau, 2005). Clearly, CVI can thus dramatically affect the child’s psychomotor functions (Costini et al., submitted) as well as the whole development (Sonksen and Dale, 2002). Although there are few studies dealing with motor development in children with CVI, clinical reports as well as experimental studies indicate that delay in development of motor skills and apparent lack of motivation to move toward or to reach or grasp objects, may relate to impaired vision, visual search or visual recognition (Fazzi et al., 2015). These latter authors have also shown, that in children with CVI, there is marked limitation of manual exploration and less spatial exploration of the environment, associated with marked delay in development of postural-motor abilities. These deficits are most severe when the damage to the brain involves the parietal lobes (Fazzi et al., 2007, 2009).

Cerebral visual impairment can also impair *visuo-motor coordination*. Impaired central and/or peripheral visual deficits naturally impair the use of vision to guide movement. The adaptive strategies made by affected children include a wide in-flight gap between fingers and thumb associated with inaccurate hand orientation, placing the whole hand down upon the target object, or gathering it up with one or both hands. These clinical signs are also seen in children with optic ataxia associated with posterior parietal pathology. In children with cerebral palsy such difficulties can be construed as motor, but the recent inclusion of CVI into definitions of cerebral palsy (Bax et al., 2007; Rosenbaum et al., 2007) aims to dispel this limited view. Typically, in children with movement deficits linked to CVI, motor performance correlates negatively with the extent to which a given task involves vision.

*Optic ataxia*, observed in cases of bilateral parietal lesions specifically affects visuo-motor coordination and hand-eye coordination. This disorder is characterized by difficulties in executing movements under visual control, especially pointing and grasping tasks (Gillen and Dutton, 2003). Affected children tend not to shift their gaze from one item to another despite other aspects of oculomotor control being evident. These two features of Balint syndrome lead to the hypothesis that although not directly ascertainable, simultanagnostic visual dysfunction is likely also to be present. Complete elimination of clutter and the presentation of a single visible toy against a plain background can lead to exploration of the surroundings and collection and study of the toy for the first time in both young (Zihl and Dutton, 2015) and older children (Little and Dutton, 2015) who have never reached out before. The features of this type of therapeutic trial resemble those of hemispatial neglect associated with motor disorders such as hypokinesia or akinesia in adults. CVI in children, particularly with hemispatial neglect (usually on the left), tend to be linked to motor neglect (see, for example: Laurent-Vannier et al., 2001, 2003, 2006) as well as to praxic deficits. Note that from a neurological point of view, it is hypothesized that in these children with dorsal stream dysfunction, the cerebral damage causes both visual impairment as well as major difficulties in gestural behavior. This association leads to a crucial question regarding the differential diagnosis between CVI and DCD (developmental coordination disorder) as we will discuss below after the definition of this disorder.

## Developmental Coordination Disorder (DCD)

### Definition

Developmental coordination disorder is a chronic neuro developmental condition that significantly impacts children’s ability to learn and perform everyday self-care and academic activities (American Psychiatric Association [APA], 2013). The occurrence of DCD in children between the ages of 5 and 11 years is estimated at between 3 and 22% worldwide (Hoare and Larkin, 1991; Wilson, 2005; Alloway and Archibald, 2008; Cardoso and Magalhães, 2009). Efforts to understand the developmental precursors of DCD as well as its clinical markers are important to avoid continued disruption to skills development, secondary impacts on self-esteem and participation, and associated issues such as obesity, poor physical fitness, and social isolation (Wilson et al., 2013). As recently underlined by Vaivre-Douret et al. (2016), there have been numerous attempts in the literature to define subtypes of DCD (Dewey and Kaplan, 1994; Miyahara and Möebs, 1995; Wright and Sugden, 1996; Macnab et al., 2001; Green et al., 2008; Vaivre-Douret et al., 2011), however, the only common features between all these profiles are difficulties in sensorimotor processes reflected by performance scores for global and fine motor skills, classified in a general DCD group. DCD has thus received considerable attention from researchers across disciplines including kinesiology, occupational therapy, pediatrics, physiotherapy, psychology, and more recently neuropsychology (Visser, 2003; Wilson and Larkin, 2008). A substantial body of literature has described the cognitive limitations of children with DCD, revealing a cognitive dysfunction profile that is attributable to an impaired information processing system including deficits in visual-perceptual and visuo-motor processing (Creavin et al., 2014), attention, planning or working memory, and learning deficits (Wilson et al., 2003; Asonitou et al., 2012; Asonitou and Koutsouki, 2016).

### Diagnosis Criteria for DCD

**Table 1** presents the DSM-5 criteria of DCD.

**Table 1:** Developmental coordination disorder (DCD): DSM-5 diagnostic criteria (American Psychiatric Association [APA], 2013).

(A) The acquisition and execution of coordinated motor skills is substantially below that expected given the individual’s chronological age and opportunity for skill learning and use. Difficulties are manifested as clumsiness (e.g., dropping or bumping into objects) as well as slowness and inaccuracy of performance of motor skills (e.g., catching an object, using scissors or cutlery, handwriting, riding a bike, or participating in sports).
(B) The motor skills deficit in Criterion A significantly and persistently interferes with activities of daily living appropriate to Chronological age (e.g., self-care and self maintenance) and impacts academic/schools productivity, prevocational and vocational activities, leisure, and play.
(C) Onset of symptoms is in the early developmental period.
(D) The motor skills deficits are not better explained by intellectual disability (intellectual developmental disorder) or visual impairment and are not attributable to a neurological condition affecting movement (e.g., cerebral palsy, muscular dystrophy, degenerative disorder).

According to criterion A, a diagnosis of DCD can be given to children who exhibit marked impairment in the development of motor skills or motor coordination in comparison to peer groups. However, as Vaivre-Douret et al. (2016) have recently pointed out, no cut-off exists regarding this criterion. In addition, this impairment in the development of motor skills is not specific to DCD since as described above, children with CVI can also demonstrate a severe delay in motor coordination compared to peer groups, due to the deleterious effects of visual impairment on motor development.

Secondly, according to criterion B, an interference with activities of daily living and impact on academic performance, prevocational and vocational activities, leisure, and play is observed in DCD children. However, as described above, the behavioral features listed under criterion A can also be observed in children with CVI.

The onset of DCD symptoms occurs in the early developmental period (criterion C), which is also the case in children with CVI.

Finally, criterion D posits that the motor skill deficits are not better explained by intellectual disability (intellectual developmental disorder) or visual impairment and are not attributable to a neurological condition affecting movement (e.g., cerebral palsy, muscular dystrophy, degenerative disorder). However, cerebral palsy can lead to both impaired motor skills and perceptual and cognitive visual impairments as we will further discuss (see for recent review Mitry et al., 2016). In addition, visual impairments do lead to motor skills deficits as discussed above. The association between praxic and visuo-spatial deficits is so frequent that some researchers have developed the concept of visuo-spatial/constructional (VSC) dyspraxia to refer to this association in children (Mazeau, 2005; Vaivre-Douret et al., 2016). However, as Costini et al. (submitted) recently discussed, diagnosing dyspraxia in children with visuo-spatial deficits is particularly difficult. This point will be addressed in the next section.

Finally, according to criterion D, the motor skill deficits are not attributable to a neurological condition affecting movement. However, as Vaivre-Douret et al. (2016) emphasize, the etiology of DCD appears confused on account of the umbrella term of motor dysfunction. Indeed, as these authors underline, minimal brain damage as well as cortical and subcortical dysfunctions have been repeatedly reported in children with DCD as we will discuss in the next section (see Investigating and Addressing CVI in Children on etiological considerations).

The conceptual overlap between DCD and the deleterious effects on motor coordination resulting from the visuo-perceptual deficits and visual deficits due to CVI needs to be addressed internationally because it is essential that children receive the most appropriate therapy regarding the nature (visual or motor) of their main deficit. The significance of the frequent association between CVI and DCD and in children is discussed below.

### CVI and DCD: What Is the Link?

Owing to CVI and fine motor disabilities being so often associated (Chokron and Démonet, 2010), there is increasing concern over the question of the differential diagnosis between CVI and DCD (Costini et al., submitted). In fact, conversely to the concept of visuo-spatial dyspraxia and as Costini et al., (submitted) propose, it is difficult to diagnose a child with DCD if he/she suffers from visual deficits, for two main reasons. Firstly, most of the tasks used to diagnose DCD involve the visual modality and secondly, as discussed above, children with CVI are at risk of a delay or deficit in motor and postural control, motor execution, oculomotor coordination, spatial orientation, representation and navigation, visual recognition and mental imagery, (for review see Lueck and Dutton, 2015). Of course, these various deficits have also been described in DCD, thus rendering enigmatic the differential diagnosis between the two conditions.

Nevertheless, given the fact that vision precedes action in the developmental course as above-mentioned, one may hypothesize that in children with visual impairments, CVI may induce DCD-like deficits while the reverse would be highly improbable.

**FIGURE 5:**
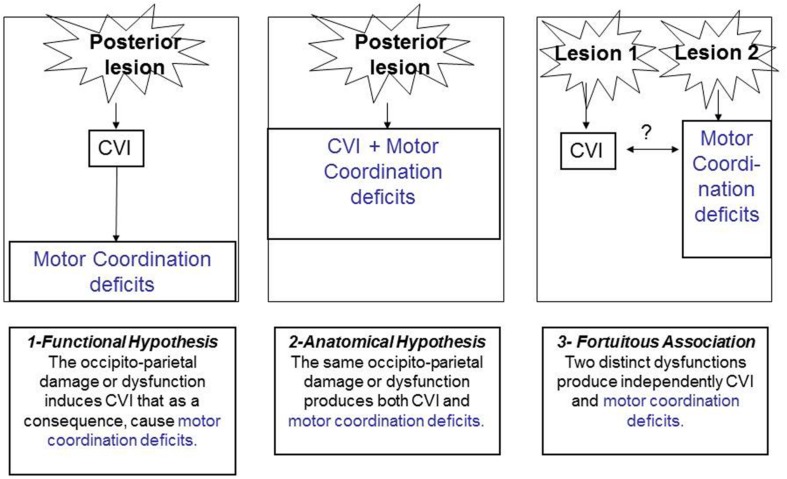
**Possible hypothetical links between CVI and motor coordination disorders**.

Indeed, as we have proposed (Gaudry et al., 2010), and as illustrated in **Figure 5**, three types of link can be invoked to explain the relationship between CVI and motor coordination deficits.

First, one could consider that there is a functional link between the two conditions. The presence of CVI will induce as a direct consequence, a delay or a deficit in motor skills, motor coordination, and motor control. Second, there could be a lesional link. Along these lines, the same brain lesion (especially along the dorsal, occipito-parietal pathway) could engender the two associated deficits. Third, there could be a fortuitous association between motor coordination deficits and CVI that could be simultaneously observed in a child but not stemming either from common brain damage or from common functional deficits. This seems highly improbable given the frequent association between the two conditions, the common aetiologies, semiology and anatomical correlates. The same line of reasoning has been proposed by Pearsall-Jones et al. (2010), regarding the link between DCD and CP.

Whatever the nature of the link between CVI and DCD, there is presently a need to inform clinical practitioners of the challenges of this differential diagnosis, and the potential for conceptual overlap. Indeed, there is an urgent need to detect and correctly diagnose CVI in children as early as possible, so as to prevent these children from potentially developing behavioral disorders (Cavézian et al., 2010; Lueck and Dutton, 2015) that may be confounded with other conditions such as DCD, or autistic spectrum disorder (Lueck and Dutton, 2015). In addition, the development of an optimal differential diagnosis between CVI and DCD would enable healthcare providers to choose the most appropriate management plan for each child and, by extension, to propose educational and parenting measures to best optimize the child’s development. It is evident that at present, a reliable diagnosis of CVI needs to be sought prior to a diagnostic label of DCD or learning disability being conceived of and applied (Lueck and Dutton, 2015).

### Neuro-Anatomical Correlates of DCD

As Pearsall-Jones et al. (2010) underline, despite the fact that DSM-5 (2013) and ICD-10 (2004), note neurological involvement as an exclusionary criterion for DCD, of the few research studies available on the etiology of DCD, most have reported neurological and preterm birth factors similar to those associated with CP. Indeed, as Vaivre-Douret et al. (2016) have recently discussed, this DSM-5 criteria do not exclude ‘minor neurological dysfunctions’ (MND), such as ‘neurological soft signs’ (NSS; Shafer et al., 1986; Hadders-Algra et al., 2010) or neuromotor disorders with mild cerebral palsy (CP). In the same way, Pearsall-Jones et al. (2010) have proposed a continuum between DCD and CP. In addition, according to several neuroimaging studies, the subcortical network of the brain could be implicated in DCD (Lundy-Ekman et al., 1991; Visser, 2003; Vaivre-Douret, 2014; Vaivre-Douret et al., 2016). Moreover, using fMRI, Zwicker et al. (2011) have proposed that in children with DCD there is an under-activation in the cerebellar–parietal and cerebellar–prefrontal networks as well as in brain regions associated with visual-spatial learning. Functional MRI studies have implicated motor regions immediately overlying the corticospinal tract (Querne et al., 2008; Kashiwagi et al., 2009; Zwicker et al., 2010, 2011). In addition, Querne et al. (2008) reported that children with DCD exhibit increased connectivity between the left middle frontal and inferior parietal cortices and reduced connectivity between the right striatum and parietal cortex. These recent connectivity studies suggest that the functional connections between the striatum and the parietal cortex, which correspond to areas integrating sensory (especially visual) information in motor responses, are altered in children with DCD (McLeod et al., 2016).

Finally, several very recent DTI studies conducted in children with DCD have reported reduced white matter integrity within the corticospinal tract (Zwicker et al., 2012) and in the superior/posterior parietal regions of the corpus callosum and the left superior longitudinal fasciculus (Langevin et al., 2014). In addition, Debrabant et al. (2016) recently demonstrated that specific white matter alterations and network topology features are associated with visual-motor deficits as well as with DCD diagnosis, thus underlining the presence of brain dysfunction in DCD.

### Aetiologies

As noted by Martin et al. (2010), a variety of neurological aetiologies have been suggested for DCD, including pre-, peri-, and post-natal complications (Kaplan and Sadock, 2007; Pearsall-Jones et al., 2009), perceptuo-motor organization (Lord and Hulme, 1988), and parietal lobe dysfunction (Wilson et al., 2004). However, as Vaivre-Douret et al. (2016) emphasize, the etiology of DCD appears confused on account of the umbrella term of motor dysfunction. Indeed, children with motor coordination difficulties are a heterogeneous group (Sumner et al., 2016) and it is difficult to ascribe a common etiology given that these children express different patterns of deficit when associated with other developmental disorders (Kaplan et al., 1998; Macnab et al., 2001). Indeed, the frequent co-morbidity between different developmental disorders (Hulme and Mackenzie, 2014) could reflect a particular vulnerability of multisensory processing abnormalities that could represent a particular risk factor in atypical development as proposed by Hill et al. (2012).

Recently, several studies have examined the comorbidity of DCD and disorders such as attention-deficit/hyperactivity disorder (ADHD), oppositional defiant disorder (ODD), conduct disorder (CD), and reading disorder (Kaplan et al., 2001; Martin et al., 2010). More specifically, the comorbidity between DCD and ADHD had been investigated and have been estimated to be around 50% (Piek and Skinner, 1999). Performing a genetic analysis, Martin et al. (2006), have recently hypothesized a strong additive genetic component to the shared etiology between ADHD and impaired fine motor ability.

In addition, Pearsall-Jones et al. (2010) proposed that DCD and cerebral palsy (CP) have similar causal pathways, and may fall on a continuum of movement disorder rather than being discrete categories. Interestingly, as above-mentioned, children with CVI often present motor, attentional, reading or learning disorders that make difficult the differential diagnosis with ADHD, CP or dyslexia. A strong argument in favor of Pearsall-Jones et al.’s (2010) hypothesis is the common aetiologies between these developmental disorders. Indeed, CP, DCD and CVI have been related to preterm birth as well as perinatal asphyxia (PA) (Vannucci and Perlman, 1997; Pearsall-Jones et al., 2009). Indeed, PA is one of the main causes of disabilities in full-term infants. According to Taniguchi and Andreasson (2008), 25% of neonates who suffered from PA develop severe and permanent neuropsychological sequelae, including mental retardation, cerebral palsy, and epilepsy. Similarly, performing a twin study, Pearsall-Jones et al. (2009) found that seven of the nine studied twins who met the criteria for DCD experienced perinatal oxygen perfusion problems. In this way and as discussed by Pearsall-Jones et al. (2010), despite the fact that DSM-5 note neurological damage as an exclusion criterion for DCD, several studies have implicated neurological and preterm birth factors similar to those associated with CP leading to the hypothesis of a continuum between DCD and CP (Pearsall-Jones et al., 2010).

As a matter of fact, as we discuss below, PA as well as preterm birth do lead to DCD, CP and CVI.

## Neurodevelopmental Consequences of PA and Preterm Birth

Indeed, in children with CVI, CP, or DCD a history of preterm birth with or without *cerebral anoxia* or *cerebral hypoxia* is often reported and if the concept of ‘soft’ neurological lesions had been used in these children, the resulting neurological signs are not ‘soft’ (Shafer et al., 1986; Jacobson and Dutton, 2000; Jacobson et al., 2006; Pagliano et al., 2007; Fazzi et al., 2009). Many studies have repeatedly brought to light evidence for specific visual as well as motor deficits in children who have survived these conditions (Carey and Gelman, 1991; Maalouf et al., 1999; Dutton and Jacobson, 2001; Atkinson and Braddick, 2007; Khetpal and Donahue, 2007; Marlow et al., 2007; Dutton and Bax, 2010; Soul and Matsuba, 2010; Birtles et al., 2012; Johansson et al., 2014; Lueck and Dutton, 2015; Azria et al., 2016). Among premature infants, especially those born between 24 and 34 weeks, the lesions, are collectively known as *periventricular white matter pathology* or PVL and are known to induce CVI (Dubowitz et al., 1980; Fedrizzi et al., 1998; Jacobson and Dutton, 2000; Jacobson et al., 2006; Fazzi et al., 2009; Dutton, 2013). Among children born at term, prolonged hypoxic-ischemic brain injury is responsible for lesions of the striate cortex, association cortices, underlying cerebral white matter, basal ganglia, thalamus and brainstem, and can also affect the oculomotor centers and lateral geniculate bodies, which together influence control over eye movement, input to the visual cortex as well as visuo-motor coordination (Dutton and Jacobson, 2001; Khetpal and Donahue, 2007; Soul and Matsuba, 2010). Furthermore, the occipital cortex can be damaged to different degrees, leading not only to cortical blindness but also profound perceptual dysfunction due to damage in the association areas. These children often have related cognitive and motor difficulties, especially of the cerebral palsy-type, making measurement of vision difficult to perform. In this way, children suffering from perceptual and motor impairments consecutive to preterm birth and/or perinatal oxygen perfusion problems can be diagnosed with either condition (CP, DCD, or CVI) regarding the relative severity of the different impairments. The presence of severe motor impairments in some of these children probably leads to an under-evaluation of visual capacities. This probably explains under-diagnosis of cortical blindness and CVI in such children. Focal lesions of early onset can be the underlying cause of cortical blindness and other forms of CVI, as can acquired posterior lesions due to stroke or cranial trauma, recognizing that shaken baby syndrome is an important cause (Mian et al., 2015). Cortical blindness can also occur—albeit less frequently—before or after resection of brain tumors, as well as a consequence of occipital cortical dysplasias, acute shunt occlusion in children with hydrocephalus and as a complication of cardiac surgery (Dutton and Bax, 2010). Other aetiologies of CVI especially of visual-field defects, in children include infections of the central nervous system, such as encephalitis and meningitis; the neurologic consequences of neonatal hypoglycemia; metabolic disorders such as mitochondrial diseases; brain malformations (e.g., holoprosencephaly, schizencephaly, or lissencephaly); and chromosomal anomalies that can be accompanied by brain malformations. Children with CVI can also manifest epilepsy of various types and severity, either in association with the above causes or as CVI due to occipital epilepsy (Dutton and Bax, 2010).

Not surprisingly, in children with DCD and CP, the same aetiologies as above-mentioned for children with CVI have been reported (see for recent reviews, Vaivre-Douret, 2014; Gomez and Sirigu, 2015; Rumajogee et al., 2016; Vaivre-Douret et al., 2016). Children with CVI, CP and DCD may thus share the same visual and visuo-motor semiology as well as the same types of etiology and there is thus a need to systematically investigate for evidence of CVI in at risk children (born preterm or in a context of cerebral hypoxia) in order to propose the most appropriate (visual or motor) rehabilitation according to the most obvious and incapacitating deficit. In the following section, we briefly present how CVI can be identified in children before other diagnostic labels, especially CP or DCD, are considered. Indeed, we propose a systematic examination of the visual function in all at risk children (born preterm or after PA) before formal learning to read (grade 1) in order to avoid the deleterious effects of CVI on motor, cognitive and social development (Jambaqué et al., 1998; Sonksen and Dale, 2002; Freeman, 2010).

## Investigating and Addressing CVI in Children

Cerebral visual impairment arises as a consequence of damage or disorder of the brain. The eyes may be affected secondarily, either owing to failure of developing normal optics (emmetropization) or as a sequel to retrograde trans-synaptic degeneration causing optic nerve atrophy or hypoplasia, and lack of retinal ganglion cells in the retina, imaged by OCT (Jacobson et al., 1997; Lennartsson et al., 2014). CVI may cause an unmeasurable or very low visual acuity in both eyes, or in cases of dorsal and/or ventral stream dysfunction, significant visual difficulties may be evident in the context of normal, or only slightly reduced visual acuities. Homonymous visual field impairment, affecting the lower visual field, or the field on one or other side, may or may not also be present. The diagnosis is made from the overall clinical picture supported by imaging and electrophysiological investigations.

### History

Parents and carers are experts in knowing and understanding their own children. In depth open history taking, allowing them to describe their child’s visual behavior provides the initial spontaneously volunteered clues to diagnosis in nearly all cases. Subsequent, non-leading structured history taking, using an inventory of questions for which expected responses cannot be deduced (Zihl and Dutton, 2015), reveals the many visual behaviors that typify the multiple patterns of CVI, but without letting parents and carers know of the expected answers. For diagnosis it is important to avoid using leading questions (Zihl and Dutton, 2015). Inventories with questions (Ortibus E. et al., 2011; Lueck and Dutton, 2015) serve an important subsequent role in profiling the visual difficulties and devising salient habilitative strategies). Interpretation of responses to questions can even lead to brain MRI scans being re-evaluated, and hitherto undetected pathology being identified (Drummond and Dutton, 2007). Indeed, as pointed out by Pearsall-Jones et al. (2010) ‘*lack of a lesion on an imaging scan does not mean that there has been no compromise or damage to brain tissue and absence of evidence is not evidence of absence.’*

### Examination of Visual Functions

*Impaired central visual functions* identified using age appropriate methodologies (Hyvärinen and Jacob, 2011; Lueck and Dutton, 2015) that are unexplained by refractive error, amblyopia or ocular disorders are highly suggestive of CVI.

As presented in **Figure 2**, *homonymous visual field disorders* can be sought in children of all ages who show evidence of giving visual attention or of missing important visual information around them.

Confrontation methods of visual field testing evaluate the child’s response to a target introduced either from behind the child, or revealed from behind an occluder in front of the child is assessed in each of the four quadrants by seeking and evaluating consistency of any resulting attentional head and eye movements, or their absence. To ensure visual fixation, the child can be asked to close his/her eyes between each trial, since the movement of the experimenter’s arm in each hemispace, presenting the visual target can induce an eye movement toward it, rendering the evaluation of the peripheral visual field impossible to perform. In addition, *the capacity to see moving targets* can be present as ‘blindsight’ in children who otherwise appear not to see, or may be diminished (dyskinetopsia) or absent (akinetopsia) (Zihl and Dutton, 2015) in those with pathology affecting the middle temporal lobes.

Formal visual field assessment by perimetry is possible in children aged over eight using classical methods, and can be performed consistently in children even less than 1 year of age, using perimetric methods that employ eye movement detection (Murray et al., 2013). A child’s inability to see the ground immediately ahead can be elicited by asking a (supported) child to raise each foot until it is visible. Peripheral lower visual field impairment can be consistently elicited in this way. In addition, the visual field can be examined very easily from the age of 4 with the *Evaluation of Vision and Attention* (EVA) battery (Chokron, 2015) if the child is able to maintain visual fixation.

### Examination of Visual Acuity and Perceptual Visual Dysfunctions

Diagnostic investigations aimed at identifying specific disabilities suggested by the history taking are chosen. (Methods that simply elicit variance from normal do not achieve this aim). Central visual functions of functional visual acuity and contrast sensitivity must be carried out to ensure that all tests performed are easily seen. Methods of evaluation that inform parents and professionals of the nature of the child’s disabilities, and thereby how to deal with him/her, are also carefully selected and judiciously employed. Children aged 4 years and older can now be examined with a battery of tests known as EVA (Cavézian et al., 2010, 2013; Chokron, 2015), aimed at identifying and treating children with CVI as soon as possible, especially before they begin primary school. Moreover, our group is currently standardizing two similar test batteries: one for infants (3 to 36 months old), and one for older children (6 to 11 years old).

Home video material provided by parents can be requested to corroborate features described on history taking, while the practitioner can also seek to reproduce the behaviors described.

*OCT imaging* of the optic nerve along with fundus photography can help identify classical optic nerve hypoplasia resulting from damage to the visual brain early during gestation (Zeki et al., 1992) and the optic nerve cupping seen in cases of marked posterior periventricular white matter pathology (Jacobson et al., 1997), on account of retrograde trans-synaptic degeneration, associated with loss of ganglion cells in the retina, also identified by OCT (Lennartsson et al., 2014).

Corroborative *MRI scanning* reveals damage affecting the visual brain from a wide range of causes, while functional MRI and tractography are beginning to show promise in specific cases (Bassi et al., 2008). However, as in 13% of children with cerebral palsy, MRI scans of the visual brain can also be normal in children with CVI (Towsley et al., 2011). Even in adults, the lesion responsible for the visual-field defect may not be found in up to 30% of the cases (Zhang et al., 2006). In this way, an MRI scan in children failing to demonstrate a visible lesion should never be considered as ‘normal’ in the presence of clinically diagnosed CVI either in children or in adults (Pearsall-Jones et al., 2010).

Normal *electroretinography* in a child with normal eyes but low vision, indicates that evidence of CVI needs to be sought using methods outlined above, while delayed low- amplitude *visual evoked potentials* with normal optic nerve examination and normal pupil reflexes, provides limited corroborative evidence of this possible diagnosis (Taylor and McCulloch, 1991; Clarke et al., 1997).

An *EEG* can prove useful for gaging parietal or occipital lesions in children with CVI. Nevertheless, we must underscore that this test is not perfectly reliable, as the EEG of children with severe impairments can still appear relatively normal. Although an EEG can be useful for locating areas of damage in the occipital lobe, it cannot be used to make a diagnosis of cortical blindness, especially in young children, whose cortical electrical activity varies widely.

## Conclusion and Perspectives

Vision has a cardinal role in a child’s visual development and CVI can compromise learning, behavioral development, and interaction with the outside world. On the other hand while DCD or CP can be the ‘visible’ deficit in a child, potential underlying CVI is invisible and often goes unnoticed by the child him or herself, who grows up unaware that their vision is defective. There is thus an urgent need for greater understanding of these impairments to enable better and earlier diagnosis and treatment, and optimal differentiation of CVI from the various neuro-developmental disorders especially DCD and CP, which share a similar semiology and common aetiologies. Indeed, as Pearsall-Jones et al. (2010) emphasize, if CP and DCD fall on a continuum of movement disorder, similar interventions as those found to ameliorate or prevent CP could be important in the treatment and prevention of DCD. The same reasoning could be applied to CVI. Regarding the frequent association between CVI, DCD and CP, it would be of interest to propose a rehabilitation program going from perceptual to motor training. Future correlation between the extensive and systematic studies of motor skills in children with visual impairments or CVI, and the visual particularities of children diagnosed with DCD or CP, is warranted to help elucidate the links and dichotomies between these clinical states. Greater knowledge and awareness of CVI in all its presentations is likely to significantly impact clinical practice and shape fundamental theories concerning visual and cognitive development in typically and atypically developing children.

## Author Contributions

All authors listed, have made substantial, direct and intellectual contribution to the work, and approved it for publication.

## Conflict of Interest Statement

The authors declare that the research was conducted in the absence of any commercial or financial relationships that could be construed as a potential conflict of interest.
